# Liquid biopsy based on small extracellular vesicles predicts chemotherapy response of canine multicentric lymphomas

**DOI:** 10.1038/s41598-020-77366-7

**Published:** 2020-11-23

**Authors:** Taismara K. Garnica, Jéssika C. C. Lesbon, Ana C. F. C. M. Ávila, Arina L. Rochetti, Oscar R. S. Matiz, Roana C. S. Ribeiro, Aline Zoppa, Adriana T. Nishiya, Mirela T. Costa, Andrigo B. de Nardi, David J. Argyle, Ricardo F. Strefezzi, Juliano C. Silveira, Heidge Fukumasu

**Affiliations:** 1 Laboratory of Comparative and Translational Oncology (LOCT), Department of Veterinary Medicine, Faculty of Animal Science and Food Engineering, University of Sao Paulo, Pirassununga, Brazil; 2 Laboratory of Molecular Morphophysiology and Development (LMMD), Department of Veterinary Medicine, Faculty of Animal Science and Food Engineering, University of Sao Paulo, Pirassununga, Brazil; 3 Clinical Veterinary Department, College of Agricultural and Veterinary Sciences, São Paulo State University ‘Júlio de Mesquita Filho’ (UNESP), Jaboticabal, SP Brazil; 4 Veterinary Hospital Anhembi Morumbi, Anhembi Morumbi University, São Paulo, SP Brazil; 5 The Roslin Institute and Royal (Dick) School of Veterinary Studies, The University of Edinburgh, Edinburgh, UK

**Keywords:** Lymphoma, Tumour biomarkers, Cancer models

## Abstract

Lymphoma is the most common type of canine hematological malignancy where the multicentric (cMCL) form accounts for 75% of all cases. The standard treatment is the CHOP chemotherapy protocols that include cyclophosphamide, doxorubicin, vincristine and prednisone, where the majority of dogs achieve complete/partial response; however, it is very important to predict non-responsive cases to improve treatment and to develop new targeted therapies. Here we evaluate a liquid biopsy approach based on serum Small Extracellular Vesicles enriched for exosomes (SEVs) to predict cMCL chemotherapy response. Nineteen dogs at the end of the 19-week chemotherapy protocol (8 Complete Response and 11 Progressive Disease) were evaluated for serum SEVs size, concentration and screened for 95 oncomirs. PD patients had higher SEVs concentration at the diagnosis than CR patients (*P* = 0.034). The ROC curve was significant for SEVs concentration to predict the response to CHOP (AUC = 0.8011, *P* = 0.0287). A potential molecular signature based on oncomirs from SEVs (caf-miR-205, caf-miR-222, caf-mir-20a and caf-miR-93) is proposed. To the best of our knowledge, this is the first study demonstrating the potential of a liquid biopsy based on SEVs and their miRNAs content to predict the outcome of chemotherapy for canine multicentric lymphomas.

## Introduction

Lymphoma is the most common type of hematological malignancy occurring in dogs. The multicentric form accounts for 75% of all canine lymphomas and is characterized by generalized lymphadenomegaly^1^. Canine lymphoma is recognized as a good model for comparative studies since it is remarkably similar to human lymphoma^2^. This cancer has a large heterogeneity with a variety of subtypes and DLBCL (diffuse large B-cell lymphoma) is the most common subtype for both species and accounts to 52% and 21%, in dogs and humans respectively^3–5^. The DLBCL probably is the most investigated tumor in veterinary medicine been relevant as spontaneous model for human DLBCL^4^. There are genetic abnormalities and mutations shared between canine and human lymphomas including PTCL activation of the PI3 kinase pathways, loss of PTEN and the tumor suppressor CDKN2, constitutive activation of the NF-kB pathway, and presence of double expressing MYC/BCL2 lymphomas^6^. The second most common subtype is PTCL-NOS (Peripheral T-cell lymphoma not otherwise specified) that is relatively uncommon in humans. However, PTCL-NOS is recognized by poor prognosis and therapeutic response in dogs and humans. This subtype is CD4-positive, often lose expression of CD5, and exhibit a histomorphology similar to that described in humans been also a candidate to translational studies^7^.

Despite the differences in diagnosis, most cases are generally treated as a single entity disease^8^. The standard protocol for canine lymphoma is CHOP protocol. The remission rates from CHOP protocol is around 73–92% and the median time of remission duration can vary widely from 5 to 12 months^9,10^. Although the initial favorable response, the majority of dogs generally relapse and the cure is rare^11,12^. The consequences of relapse are a decrease in chances to achieve remission again and the response to secondary protocols^13^. Dogs that do not show an initial response to CHOP or relapse during or after chemotherapy regimen are a source of information about refractory profile and chemoresistance development^14,15^. However, to determine which patients will respond or not to CHOP before starting the chemotherapy is a major challenge in canine lymphoma.

Liquid biopsies are gaining attention to monitor and identify therapeutic response in human’s hematological malignancies. The search for molecules in blood such as tumor cDNA, exosomes or miRNAs can improve the molecular pathogenesis of lymphoma and also bring relevant information to help clinical and treatment decision. Exosomes are small extracellular vesicle (30–150 nm) with double lipidic membrane and can be involved in cellular communication as well as transporting of important biological molecules (mRNA, miRNA, metabolites, proteins, receptors) between cells^16^. These vesicles are also enrolled in cancer development and can be easily detect in organic fluids such as blood, urine, saliva^17,18^, thus eliciting exosomes as potential candidates for liquid biopsy approaches^19^. MicroRNAs are small non-coding RNA molecules responsible for post-transcription regulation and can be carried by exosomes regulating important to pathways related to cancer metastasis, prognosis, therapeutic response and chemoresistance mechanisms^20–22^. Karlee and collaborators analyzed 38 miRNAs and found altered expression of miR-127, miR-34a and miR125b in plasma comparing dogs with lymphoma that relapsed and healthy control dogs^23^. An in vitro study showed three exosomal miRNAs (miR-151, miR-8908a-3p, and miR-486) and CD82 protein with different expression between vincristine-sensitive canine cancer cell lines (CLBL-1 and GL-1) and the resistant cell line (UL-1)^24^.

Although advances have been made in the field of veterinary oncology there is no test to predict the therapeutic response in canine lymphoma to the best of our knowledge. Therefore, our goal was to evaluate the potential of SEVs and its miRNAs content as predictive marker for therapeutic response and outcome of canine multicentric lymphoma.

## Results

### Patient and control groups

A total of 19 dogs with multicentric lymphadenopathy were cytologically diagnosed with lymphoma and other 30 healthy dogs were used as controls. Age average was 2.43 years (range 0.5–7 years) and 7.8 years (range 3–11 years) in control and lymphoma group, respectively, and as expected, this difference is significant (*P* < 0.0001). All other relevant characteristics from animals of both groups can be found in supplementary table 2. All the 19 cases had the diagnosis of large-cell lymphoma by cytology^25^. Only 7 dogs had additional information about diagnosis (immunohistochemistry and/or PARR) being 5 dogs diagnosed as DLBCL by immunohistochemistry^26^ and 2 diagnosed as B-cell lymphoma by clonality test PARR^27^ (Supplementary table 3). The observation period for lymphoma patients comprehended 223 to 837 days after the end of chemotherapy protocol and data were recorded from January 2017 to February 2020.

**Figure 1:**
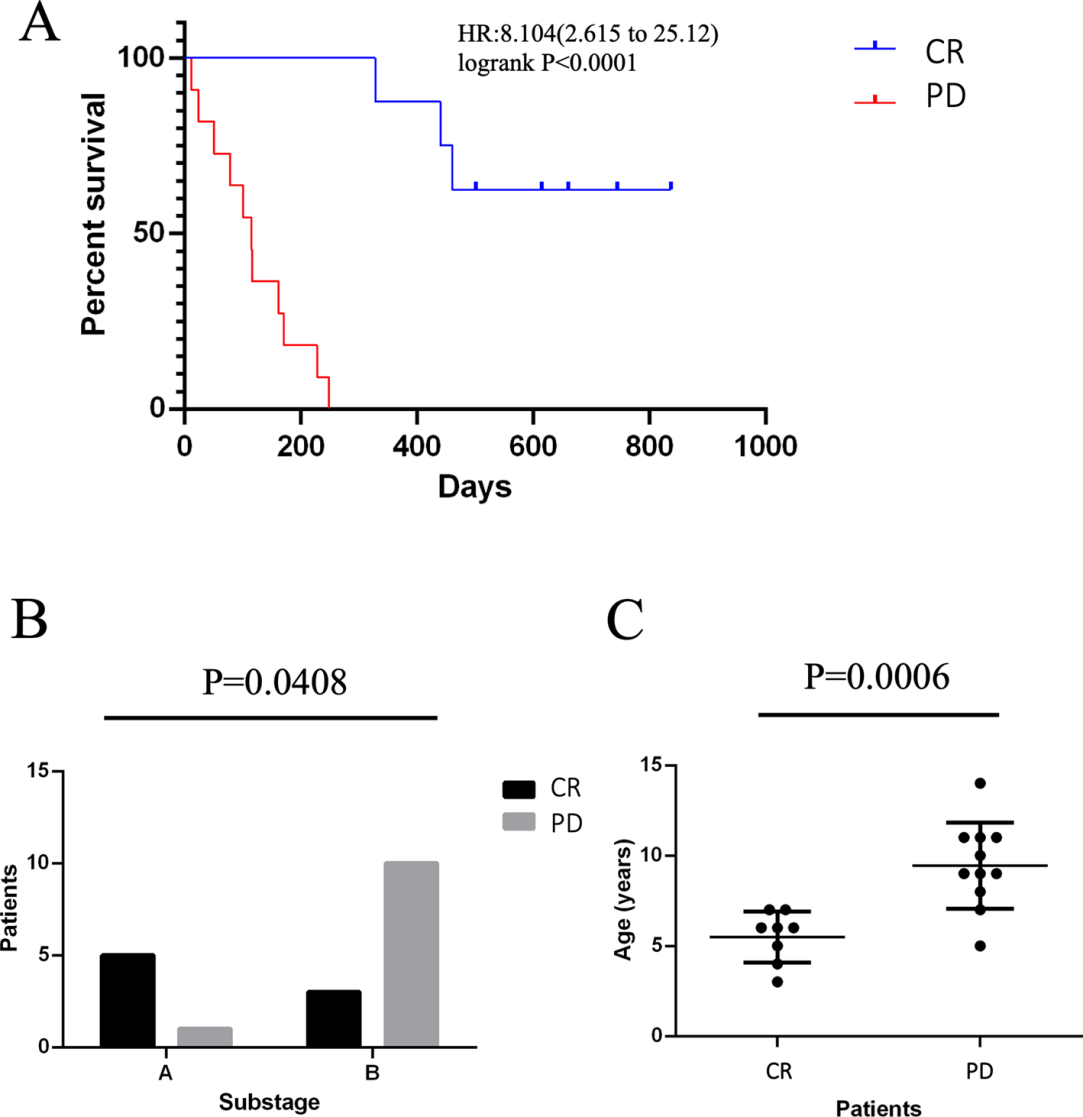
Therapeutic response of lymphoma patients and clinical differences between the group of complete response (CR) and the group of progressive disease (PD). (**A**) Survival proportions of groups CR and PD (*P* < 0.0001), Censored patients (alive dogs) are indicated on the Kaplan–Meier curve as tick. (**B**) Frequency of substages A and B in CR and PD groups (*P* = 0.0408). (**C**) Mean age between groups CR and PD (*P* = 0.0006).

All the 19 patients with lymphoma were treated with CHOP protocol. The evaluation of therapeutic response showed that 8/19 (42%) patients achieved complete response at the end of the protocol. Eleven patients (11/19, 58%) did not respond to chemotherapy or relapsed prior to completion of the 19-week protocol. Overall survival was significantly different between CR and PD groups (*P* < 0.0001, Fig. 1A) with median survival time of 124 and 573 days, respectively for PD and CR groups.

In addition, CR and PD groups were different for sub-stage (*P* = 0.0408, Fig. 1B) and age (*P* = 0.0006, Fig. 1C), where the patients with progressive disease presented significantly more systemic signs and are older than CR patients.

### Characterization of the SEVs isolated from canine serum

**Figure 2:**
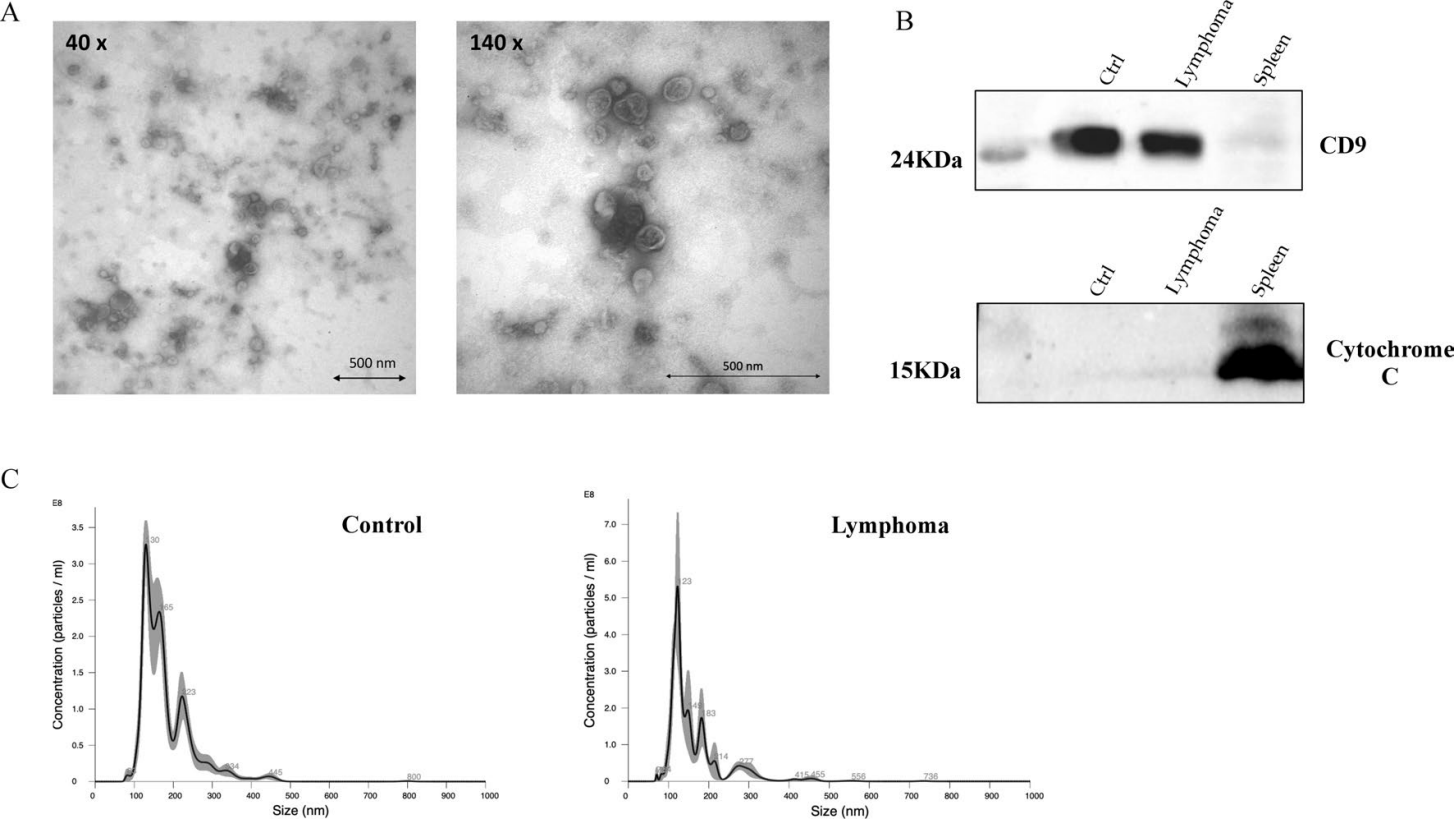
Characterization of SEVs isolated from canine serum. (**A**) Transmission electron microscopy showing SEVs with lipid bilayer (scale bar 500 nm). (**B**) Western blotting analysis of proteins CD9 and Cytochrome C in SEVs and canine tissue (spleen). The CD9, a transmembrane protein from tetraspanin family, was detected in SEVs from canine serum (control and lymphoma) and tissue (spleen). The Cytochrome C, a mitochondrial marker, was only detected in spleen, thus confirming the absence of cell contamination in exosome samples. The images were obtained with ChemiDoc MP Image System (Bio-Rad; Hercules; CA, USA), analysis was performed using the Bio-Rad Image Lab 6.0.1 (Bio-Rad; Hercules; CA, USA) and processed using the GIMP 2.10.14 ’’https://www.gimp.org’’) full-length blots/gels are presented in Supplementary Figure 1. (**C**) Representative NTA analysis of the particle size (nm) of SEVs isolated from control and lymphoma group showed particles around 30–150 nm in diameter, performed through NanoSight.

SEVs isolated from the serum of control animals and lymphoma patients had a typical “donut-like” appearance by transmission electron microscopy (Fig. 2A). The NTA analysis showed a range of diameter between 30 and 150 nm agreeing to literature^28^ (Fig. 2C). SEVs from representative cases (1 case from lymphoma group and 1 from control group) were positive for CD9 and negative for Cytochrome C whereas the spleen sample had less expression of CD9 and was positive for Cyt-C (Fig. 2B and supplementary Figure 1). No difference was found on concentration and size of SEVs in sera from lymphoma and control groups.

### The concentration of serum SEVs at diagnosis predicts chemotherapy response

**Figure 3:**
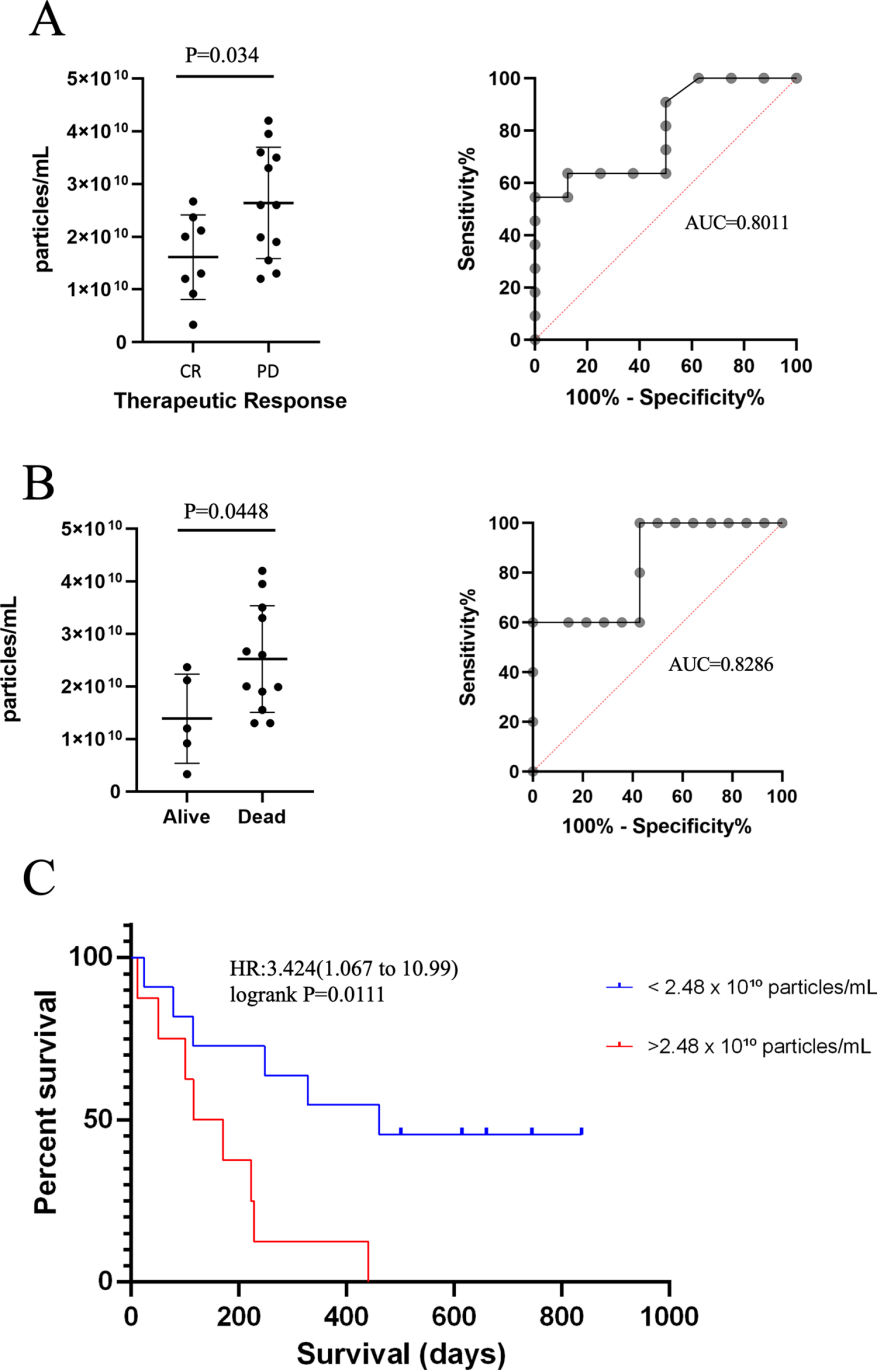
Serum SEVs at the diagnosis predicted the response to chemotherapy. (**A**) Higher concentration of SEVs in PD group when compared to CR group (*P* = 0.034) and ROC curve using the concentration of SEVs for therapeutic response (AUC = 0.8011 and *P* = 0.0287). (**B**) Higher concentration of SEVs in dogs that died due lymphoma compared to dogs that stayed alive (*P* = 0.0448) and ROC curve for survival (AUC = 0.8286 and *P* = 0.0332). (**C**) Kaplan Meier survival analysis for SEVs concentration with 2.48 × 10^10^ particles/ml cut-off point (log rank *P* = 0.0111).

Nineteen dogs (8 CR and 11 PD) were evaluated to test the predictive and prognostic value of the size and concentration of SEVs. Patients that had disease in progression presented higher concentration of serum SEVs at the moment of tumor diagnosis than patients with complete response (*P* = 0.034 Fig. 3A). The ROC curve analysis was significant and the Area Under the Curve (AUC) for concentration of SEVs to predict the response to CHOP was 0.8011 (*P* = 0.0287, Fig. 3A). Patients who died due lymphoma had higher concentration of SEVs at moment of tumor diagnosis than patients that stayed alive (*P* = 0.0448, Fig. 3B). The ROC curve analysis showed that SEVs concentration was significant to predict survival (AUC = 0.8286, *P* = 0.0332). Kaplan Meier survival analysis using SEVs concentration at D0 showed that dogs with > 2.48 × 10^10^particles/ml lived 318 days less compared to dogs with < 2.48 × 10^10^particles/ml, 143 vs 461 days respectively (*P* = 0.0111, Fig. 3C). The size of the SEVs was not predictive or prognostic for lymphoma patients (*P* = 0.1233).

The therapeutic response (CR or PD) was inversely associated with concentration of SEVs (Spearman r = − 0.516, *P* = 0.024), age (r = − 0.735, *P* = 3.34 × 10^–4^) and sub-stage (r = − 0.567, *P* = 0.011). There was no effect of age (*P* = 0.5853), stage (*P* = 0.7532) and substage (*P* = 0.4142) on SEV concentration. Thus, we performed a multiple linear regression to evaluate the predictive power of SEVs on therapeutic response including the concentration of SEVs, age and substage as independent variables. The model is highly significant to predict the therapeutic response (R^2^ = 0.7789, *P* < 0.0001). Then, we also performed the analysis excluding the SEVs concentration (R^2^ = 0.6746, *P* = 0.0001), which indicated that the use of SEVs along with other information from the patient should be considered.

### Oncomirs analysis revealed potential markers for clinical response

**Figure 4:**
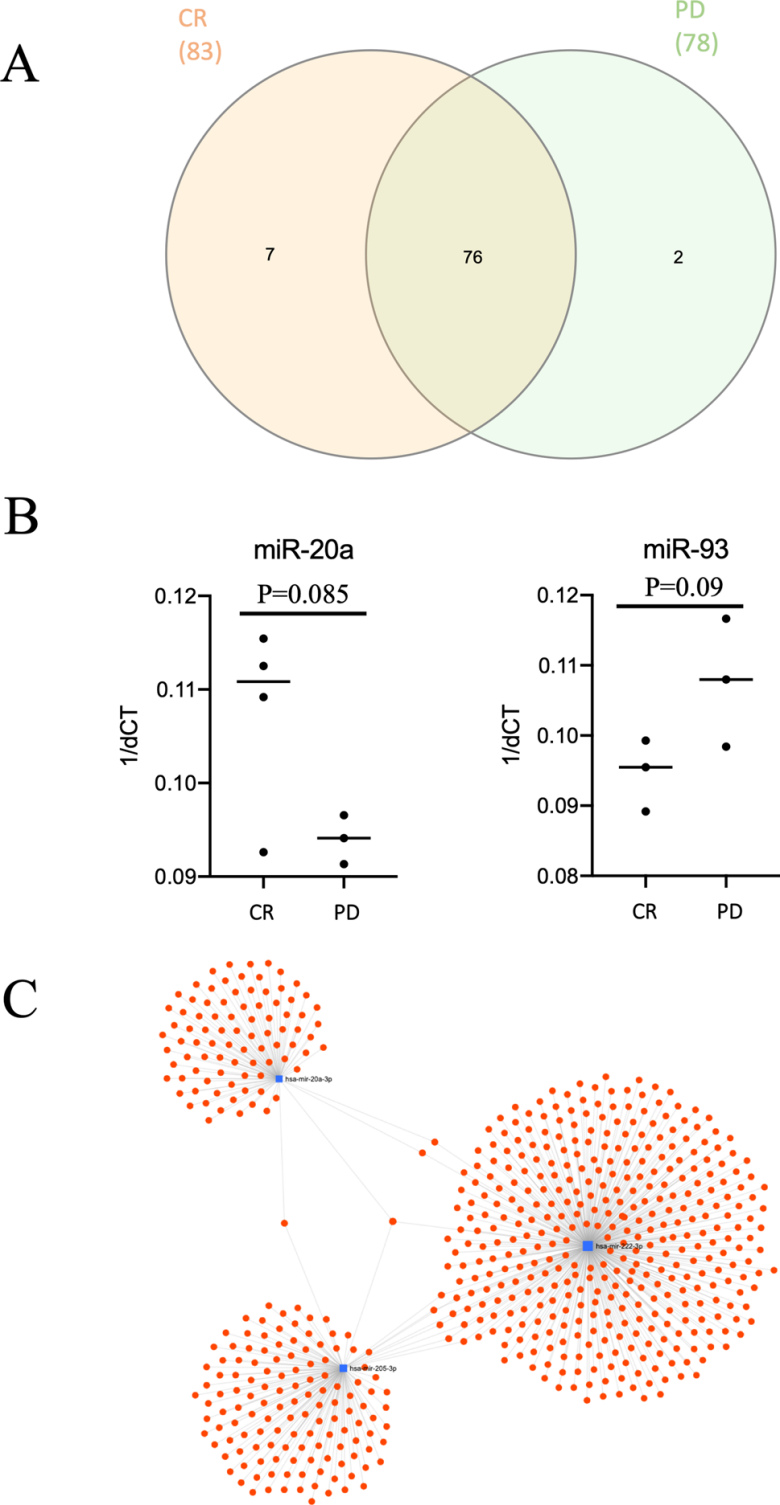
Screening of exosomal oncomirs revealed potential markers for clinical response. (**A**) Venn diagram of 85 oncomirs found to be expressed in SEVs. (**B**) Expression of mir-20a and mir-93 is significantly different between groups CR and PD. (**C**) Target genes from the oncomirs associated with complete response lymphoma patients. It is possible to notice few genes targeted for more than one oncomir.

The screening of 95 oncomirs in exosome samples (5 from CR and 5 from PD groups) showed that 85 oncomirs were detected in at least one patient (Fig. 4A). In addition, 76 oncomirs were identified in both groups, 7 were found only in the CR group (miR-151-5p, miR-190a, miR-200c, miR-204, miR-488, miR-183, miR-205) and two were found only in PD group (miR-196a, miR-10b, Fig. 4A). Statistical analysis showed 2 oncomirs with higher frequency in the CR group: miR-205 (3/5 vs. 0/5, *P* = 0.0384) and miR-222 (4/5 vs. 1/5, *P* = 0.0578, Fig. 4B). When we compared the expression level, 2 oncomirs were suggestive (*P* < 0.10) for differentially expressed between CR and PD groups. The mir-20a was more abundant in patients with CR (*P* = 0.085, Fig. 4), while miR-93 in patients with PD (*P* = 0.09, Fig. 4B). The pathway analysis performed for oncomirs found in the CR group (miR-20a, miR-205 and miR-222, Fig. 4C) enriched for pathways involved in activation of BH3-only proteins (*P* = 0.009), PIP3 activating Akt signaling (*P* = 0.018) and signaling by SCF-kit (*P* = 0.018, Table 1).

**Table 1:** Enriched pathways for oncomirs in the complete response group.

Pathway	Expected	Hits	*P* value
Gene expression	32.2	65	1.13e−05
Activation of BH3-only proteins	0.869	7	0.00941
BH3-only proteins associate with and inactivate anti-apoptotic BCL-2 members	0.302	4	0.018
PI3K events in ERBB4 signaling	3.55	12	0.018
PIP3 activates AKT signaling	3.55	12	0.018
Signaling by SCF-KIT	5.03	15	0.018
PI3K events in ERBB2 signaling	3.55	12	0.018
Cellular responses to stress	9.67	23	0.018
Oncogene Induced Senescence	1.13	7	0.018
Role of LAT2/NTAL/LAB on calcium mobilization	3.89	13	0.018
PI-3K cascade:FGFR2	3.55	12	0.018
PI-3K cascade:FGFR3	3.55	12	0.018
PI-3K cascade:FGFR4	3.55	12	0.018
PI-3K cascade:FGFR5	3.55	12	0.018
PI3K/AKT activation	3.66	12	0.0227
GAB1 signalosome	3.7	12	0.0227
Cellular senescence	5.4	15	0.0227
Intrinsic pathway for apoptosis	1.36	7	0.0234
Cell cycle	18.8	34	0.0325

## Discussion

The chemoresistance and relapse are events commonly observed during the treatment of canine lymphomas being directly related to therapeutic efficacy and survival^29^. Data from the literature show that 85% of dogs with lymphoma treated with CHOP 19 weeks protocol, achieves complete or partial response^30^. However, approximately 50% of dogs relapse the disease within 1 year after diagnosis^1,29,31^. The relapse decreases the chances of achieve remission again and respond to secondary protocols^13^. Thus, the ability to predict the cases that will not respond to CHOP are highly needed. In this work, we show that a liquid biopsy based on SEVs of patients with multicentric lymphoma can predict the response to CHOP protocol an accuracy of 80% alone or 78% in a multivariate model. Also, we found exosomal oncomirs associated with response to CHOP protocol that can be used to improve the ability to predict the response to treatment.

In our study, lymphoma patients that didn’t respond to chemotherapy had progressive disease and significant short overall survival. We found increased concentration of serum SEVs at the diagnosis in these patients when comparing to patients that had complete response. However, there was no difference between lymphoma patients and control patients regarding exosome concentration. At a first glance, one may consider this a setback, but we shall not forget that the major need nowadays is to predict response to chemotherapy and survival and not to diagnoses lymphoma which can be done easily by the clinician. In addition, prediction of therapeutic response based only in clinical signs and lymph node measurement can result in misdiagnosis of complete response^31–34^, corroborating the need of more quantitative markers to help evaluate therapeutic response and improve canine lymphoma treatment.

We also found that age at the diagnosis and the presence of systemic symptoms (sub-stage B) are related to shorter overall survival and both are significant for prediction of therapeutic response. The substage B is a well-known prognostic factor for response to chemotherapy of canine lymphomas^35,36^. No other clinical data was associated with therapeutic response, and since we didn’t have immunophenotyping or histopathological analysis for all patients we didn’t consider their possible association with therapeutic response. A study conducted by Moore and colleagues found that anemia and high body weight in elderly dogs associated to poor prognosis in lymphoma patients^37^, corroborating the data in our study since 10 out 11 dogs from PD group have systemic signs (sub-stage B). Interestingly, the multivariate analysis using the concentration of SEVs, substage and age improved significantly (*P* < 0.0001) the predictive power to 0.779 (R^2^). In humans, the quantification of blood exosomes is a potential prognostic marker for esophageal squamous cell carcinoma^38^ and patients with high levels of exosomes in plasma tended to have shorter overall survival than patients with low levels, corroborating our results^39^.

Our screening of 95 oncomirs in blood SEVs revealed 4 possible biomarkers related to the outcome of lymphoma patients: mir-205 was found exclusively in the complete response group; mir-222 have a significant higher frequency in CR group; mir-20a was more expressed in the group CR and only mir-93 was more expressed in the PD group. The functional enrichment analysis of miR-205, miR-222 and miR-20a in CR group showed involvement in activation of BH3-only proteins (*P* = 0.009), PIP3 activates AKT signaling (*P* = 0.018) and signaling by SCF-kit (*P* = 0.018). The BH-3 only group of pro-apoptotic Bcl-2 proteins consists of Bim (BCL2L11), Puma/BBC3, Bad (Bcl-2/Bcl-x-associated death promoter), Bid (BH-3 interacting-domain death agonist), Bik (Bcl-2-interacting killer), Noxa/PMAIP1, Bmf (Bcl-2-modifying factor), and Hrk (Harakiri) and are essential for initiating the apoptotic cascade. Loss of BH3-only proteins is involved in B-cell lymphomagenesis^40^. The PI3K/AKT/mTOR is an important pathway in cancer related with competitive growth, survival, increase metastatic ability and resistance to conventional therapy and also has been explored for canine oncology^41,42^. The whole genome and exosome sequencing show B-cell and T-cell lymphoma mutated genes related to dysregulation of the PI3k/PTEN signaling axis in canine lymphomas^43,44^. The inhibitor of PI3Kδ is on phase I/II for canine spontaneous lymphoma as a promising therapy for recurrent and refractory cases^45^. SCF-kit is the complex composed by ligand SCF and tyrosine kinase receptor c-Kit. The gene c-Kit is a proto-oncogene that is subject to dysregulation and gain-of-function mutations and amplifications that promote tumorigenesis in a variety of tumor types^46^. However, c-Kit can also promote apoptosis and inhibit tumor growth in absence of SCF (stem cell factor)^47^.

The miR-205 was exclusively found in CR group. This miRNA can act as a tumor suppressor or oncogene depending on specific cancer context or its target genes^48^. There is no information in literature about the expression of miR-205 in dogs with lymphoma, but in humans low expression level of this miRNA is associated with B-cell lymphomas^49^. The miR-222 was higher expressed in CR group and was associated with good prognosis. Contradictory results recently published showed miR-222 expression in plasma of dogs with B-cell or T-cell lymphoma was negatively correlated with OS and PFS^23^. The miR-20a was more expressed in CR group and were related to a good prognosis. The miR-20a is a member of mir-17-92 cluster and is found expressed in B-cell lymphomas and B-cell chronic lymphocytic leukemia in humans^50^. The plasma levels of miR-20a was associated with high mortality in humans with DLBCL^51^. In dogs, there is one report showing increased miR-20a in plasma of T-cell lymphoma patients in comparison to control animals^34^. At last, we found only the miR-93 being more expressed in PD group. The miR-93 is a precursor of miR-17 family and its expression is associate to aggressive cancer phenotypes due increasing metastasis, tumor growth, invasion and angiogenesis in different human cancer as NSCLC, ovarian cancer, gastric cancer and glioma^52–55^. Khare et al.^51^ showed that miR-93 is downregulated in plasma of humans diagnosed with Hodgkin lymphoma (HL), and it is also associated high mortality rate in DLBCL, supporting our results of exosomal increased levels of miR-93 in non-responder’s group. Taken together, these data suggest a possible molecular signature for SEVs content based on oncomirs related to the outcome of lymphoma patients, but additional experiments are needed to validate these findings using more samples. In fact, other studies already showed the potential of microRNAs as potential biomarkers in canine cancers^23,56^.

One of the challenges of our study was to find multicentric lymphoma cases that fit in proposed inclusion criteria. Lymphoma has higher incidence in older dogs (> 10 years) which increases the odds of concomitant diseases. Other challenge was to find patients with complete information about diagnosis, immunohistochemistry and histopathology. This is explained by the fact that some clinicians consider the therapeutic approach soon after the cytologic diagnosis, not recommending the biopsy for further histopathology/immunophenotyping analyses. A study conducted by Regan and colleagues during the 2009 Veterinary Cancer Society Annual Conference interviewed 519 clinicians and showed that 76% of volunteers recommended the immunophenotyping but only 28% recommended the lymph node histopathology for full staging^12^. Probably in our field conditions (Brazil) these numbers are even lower mostly due to the price of the diagnostic tests.

To the best of our knowledge, this is the first study to show the potential of predicting response to CHOP and outcome of lymphoma patients using a liquid biopsy based on SEVs and their miRNAs content. There are two studies recently published involving molecular markers for prediction. Parissenti by et al.^33^ revealed that RNA disruption in affected lymph nodes can be associated with good response to CHOP chemotherapy. Other study evaluated the miRNAs concentration in plasma and identify 8 miRNAs had differential expression in the non-remission group compared to dogs that completed CHOP in complete response^23^. Regardless these findings, the literature about predictive markers for canine multicentric lymphoma continues limited.

In conclusion, data presented in this work will advance veterinary oncology by providing evidence for the future use of liquid biopsies to assist clinicians to early identify refractory patients and help to guide the best treatment possible. The validation of the oncomir’s signature of lymphoma patients who didn’t respond to chemotherapy will have the potential to guide future studies on targeted therapies opening a window to precision medicine in veterinary oncology. Although the future use of this technique in daily routine might demand specific equipment as nanoparticle tracking analyzers, there are new approaches for exosome capture and quantification by ELISA for example, which will allow the worldwide use in the future.

## Material and methods

### Case recruitment

All animal work was conducted in accordance with a protocol approved by the Ethic Committee on Animal Use of the Faculty Animal Science and Food Engineering of the University of Sao Paulo (protocol number CEUA-FZEA-USP/9827200717). All animal procedures were carried out following the CEUA-FZEA-USP guidelines and informed consent was obtained from all owners in a specific protocol approved by the Ethic Committee on Animal Use of the Faculty Animal Science and Food Engineering of the University of Sao Paulo (protocol number CEUA/9827200717). Thirty healthy dogs were selected to compose the control group and nineteen dogs diagnosed with multicentric lymphoma were selected according to eligible criteria. The samples were collected from January 2017 to January 2019. The control group samples were provided by Veterinary Hospital of Faculty of Animal Science and Food Engineering in Pirassununga (Pirassununga, SP, Brazil). Lymphoma samples were collected from two other veterinary hospitals: Anhembi Morumbi Veterinary Hospital in São Paulo (São Paulo, SP, Brazil) and Governor “Laudo Natel” Veterinary Hospital in Jaboticabal (Jaboticabal, SP, Brazil). The control group was composed of prospective and retrospective serum samples from healthy dogs tested by clinical examination and confirmed by blood tests (complete blood count, alanine aminotransferase, creatinine, alkaline phosphatase, urea), without previous diagnosis of any disease (cancer, chronic inflammation, metabolic disease, reproductive disease, obesity and others) and with updated vaccination and deworming. Lymphoma samples were from prospective and retrospective cases of dogs diagnosed with multicentric lymphoma by cytological analysis. The cases were classified according to published by WHO [clinical examination, cytology, histopathology, PARR (PCR for antigen receptor gene rearrangements), immunohistochemistry]. The staging followed the criteria published by WHO using clinical examination, complete blood test, abdominal ultrasound, thoracic radiography and when possible the bone marrow analysis^57^. Only patients without previous diagnosis of cancer, treated with CHOP protocol, without concomitant diseases and did not receiving other drugs beside CHOP were selected to participate of this study.

### Evaluation of therapeutic response

The response to the chemotherapy was evaluated following the criteria published before^58^. Evaluation of therapeutic response was made at 19th week (at the end of protocol) for dogs who completed the protocol and for dogs who died during the treatment the last therapeutic response was considered. In addiction when available we also recorded clinical information about returns.

### Serum samples, isolation and characterization of SEVs

Serum samples were collected at the diagnosis (D0) before the start of the CHOP chemotherapy protocol and for control group during the clinical examination. SEVs were obtained from samples of D0 from lymphoma patients and control group. Upon collection, 2 ml of serum was centrifuged at 4 °C in order to remove live cells (300×*g* for 10 min), cellular debris (2000×*g* for 10 min) and large extracellular vesicles such as microvesicles (16,500×*g* for 30 min). The remaining supernatant was divided in 200 µl aliquots and maintained at − 80 °C until isolation of SEVs. On the day of use, 200 µl of serum was filtered through a 0.20 μM sterile syringe filter (PES membrane; KASVI) in order to remove any remaining large EVs. Finally, this fluid was centrifuged twice at 120,000×*g* for 70 min (Optima XE-90 Ultracentrifuge; rotor 70 Ti; Beckman Coulter) in order to isolate SEVs as previously described^28^. The supernatant was discarded, and the exosome pellets were resuspended in 50 μL of phosphate buffered saline (1 × Ca^2+^/Mg^2+^ free PBS; 137 mM NaCl, 2.7 mM KCl, 10 mM Na_2_HPO_4_, 2 mM KH_2_PO_4_) until further use. SEVs isolated from canine serum were characterized based on their morphology and size using transmission electron microscopy; by the presence of specific membrane proteins CD9 and absence of Cytochrome C using western blotting. Particle size and concentration was determined using nanoparticle tracking analysis (NTA). The protocols were based on previous work and briefly described above^59,60^.

### Transmission electron microscopy

The transmission electron microscopy was performed according Avila and colleagues^60^. Briefly, exosome pellets isolated from 200 µl of serum of dogs from control and lymphoma group were diluted in 50 μl of fixation solution (0.1 M cacodylate; 2.5% glutaraldehyde and 4% paraformaldehyde at pH 7.2–7.4) for 2 h at 4℃. Subsequently, SEVs were diluted in 2 mL of 1X Ca^2+^/Mg^2+^ free PBS, and the solution was centrifuged once in order to obtain pellets of SEVs (120,000×*g*, 70 min, 4 °C). The pellet was diluted in 100 μL of milli-Q water and placed in a copper grid for 20 min at room temperature in order for it to dry before staining. The grid was inserted into 2% of uranyl acetate and then analyzed using a transmission electron microscope (FEI Tecnai 20; LAB6 emission; 200 kV).

### Nanoparticle tracking analysis

The nanoparticle tracking analysis was performed according Avila and colleagues^60^ with minor modifications. Briefly, the SEVs isolated from 200 μL of serum from control and lymphoma group samples were resuspended in 50 μL of 1X Ca^2+^/Mg^2+^ free PBS. The particle size and concentrations were measured using Nanosight (NS300; NTA 3.1 Build 3.1.45, Malvern). The dilution factor was between (1:100 and 1:200) for 1 × Ca^2+^/Mg^2+^ free PBS depending on sample concentration. The analysis was performed by capturing 5 videos of 30 s each, using a sCMOS camera at camera level 14 and under a controlled temperature of 37 °C. A threshold of 5 and a total valid track up to 2.500 were considered in this analysis.

### Western blotting

The protein lysate from SEVs were obtained using RIPA buffer and 0.1% proteinase inhibitor cocktail (Halt Protease Inhibitor Cocktail (100 ×), Thermo Fisher Scientific, USA). Canine tissue (spleen) collected from a necropsy was used as negative control. The samples were prepared using 5 μl laemmli buffer and beta-mercaptoethanol 4 × (Bio-rad) in 20 μL (~ 20 μg) of protein solution. Denaturation of the proteins was accomplished by incubation at 95 °C for 5 min. Samples were then loaded onto SDS-PAGE 12% polyacrylamide gel, separated by 100 V for 140 min electrophoresis and transferred on to a PVDF membrane (1704156; Trans-Blot Turbo; Bio-Rad; Hercules; CA, USA). The transference was performed at 80 V for 120 min and then, the membrane was washed in 1 × Tris buffered saline with Tween-20 (TBST) and maintained in a blocking buffer (5% of bovine serum albumin (BSA) in TBST at room temperature for 1 h. After that, the membrane was incubated overnight with a primary antibody at 4 °C. The proteins CD9 and Cytochrome C were evaluated using antibodies CD9 (C-4) Santa Cruz (sc-13118) dilution (1:2000) and Cytochrome C (C-20) Santa Cruz (sc-8385) dilution (1:750). After incubation, the membrane was washed three times using 1 × TBST for 5 min each and then incubated with secondary anti-mouse (1:2000; #7076S; Cell Signaling Technology) and anti-goat (1:2000, #B2709; Santa Cruz Biotechnology) for 1 h at room temperature, both antibodies were conjugated to horseradish peroxidase (HRP) for chemiluminescent detection. Finally, the membrane was washed three times using 1 × TBST and exposed to a detection solution (170-5060; Clarity Western ECL). The images were obtained with ChemiDoc MP Image System (Bio-Rad; Hercules; CA, USA), analysis was performed using the Bio-Rad Image Lab 6.0.1 (Bio-Rad; Hercules; CA, USA).

### RNA extraction

RNA was obtained using TRIZOL (Life Technologies, USA) with previous addiction of coprecipitate PolyAcryl Carrier (PC152, MRC, USA) from 5 samples of responder’s group and 5 samples non-responder’s group. For this assay, 8 μL of PolyAcryl and 750 μL of Trizol in 50 μl were added to exosome samples and incubated for 5 min. Then, 200 μL of chloroform were added and incubated for 3 min. The samples were centrifuged for 15 min as 12,000×*g* at 4 °C. The aqueous phase was mixture with 500 μL of isopropanol and incubated for 10 min. The samples were centrifuged for 10 min at 20,000×*g* at 4 °C. The pellet was resuspended in 1 mL of 75% ethanol and centrifuged for 5 min at 20,000×*g* at 4 °C twice. Finally, the supernatant was discarded, and the pellet was resuspended in 10 μL of RNase-free water. Samples were treated using DNAse kit (Ambion, USA) according to manufacture instructions and stored in -80° freezer until use. RNA concentration and quality were assessed by NanoDrop (Thermo Fisher Scientific, USA) and only samples which presented values of A260/A280 between 1.7 and 1.9 were used.

### Real-time PCR for oncomirs

Total RNA including miRNAs were reverse transcribed using miScript II RT Kit (Qiagen, USA) according to the manufacturer’s instructions. Briefly, 10 μL reactions were made, containing 50 ng of total RNA, 10 × miScript Nucleic mix, nuclease-free water, miScript reverse transcriptase and 5 × miScript HiFlex Buffer, in accordance with the manufacturer’s instructions. The reaction was incubated at 37 °C for 60 min followed by 95 °C for 5 min. Quantitative RT-PCR was performed using miScript SYBR Green PCR Kit (Qiagen, USA). The total volume of the reaction mixture was 6 μL and contained 2 × Power SYBR Green PCR Master Mix (Thermo Fisher Scientific, USA), 10 × miScript Universal Primer, nuclease-free water, 0.2 ng of cDNA and 1 μL of specific forward primer which was designed based on canine mature miRNA sequences and according to mirBase database (https://www.mirbase.org, Supplementary Material Table 1). A total of 95 miRNA sequences were evaluated. Amplifications were performed using QuantStudio 6 Flex (Thermo Fisher Scientific, USA). The reactions were exposed to 95 °C for 15 min, followed by 45 cycles for 15 s at 94 °C, 30 s at 55 °C and 30 s at 70 °C. This was followed by melting curve according to the manufacturer’s instructions. We considered the miRNA present when cycle threshold (CT) was less than 37 cycles in at least three biological repetitions with adequate melting curves. Upon confirmation, CT was normalized using the geometric means of miR-99b, Hm/Ms/Rt T1 sRNA and RNU43snoRNA^59^. Data analyses were performed to evaluate the miRNAs that were described as common, exclusive as well as differently abundant in CR and PD groups. The analysis of the Oncomirs was carried out in three stages, aiming answer the questions: how many miRNAs were expressed by each group? Were there exclusive miRNAs in the groups? Was there any miRNA that was differently expressed between the groups? First step was the frequency analysis, and the miRNA was considered positive in the group when we had his expression in at least one sample. Second was to investigate exclusive Oncomirs related to CR and/or PD using statistical test of frequency, and third was analyze the difference in Oncomirs expression between CR and PD (*P* < 0.1). Pathway enrichment analysis was performed with miRNet using a hypergeometric test for reactome pathways and significant results were considered when *P* < 0.05^61^.

### Statistical analysis

All data were evaluated for homoscedasticity with D’Agostino & Pearson normality test and parametrical or non-parametrical tests were chosen accordingly. For two group comparisons unpaired T test or Mann–Whitney were used; contingency tables were analyzed with Fisher’s exact test; survival curves were analyzed with Kaplan–Meier method followed by Log-rank (Mantel-Cox) test; correlation was performed with Pearson or Spearman r tests; ROC curves were used for the predictive value; and multiple logistic regression was used for multivariate analysis. *P* values were considered significant when < 0.05 and suggestive when *P* < 0.10.

## Supplementary information

Supplementary Tables.

## Data Availability

The datasets generated during and/or analyzed during the current study are available from the corresponding author on reasonable request.
