# Supplementary material for: Liquid biopsy based on small extracellular vesicles predicts chemotherapy response of canine multicentric lymphomas
Source: Sci Rep. 2020 Nov 23;10:20371. doi: 10.1038/s41598-020-77366-7 (PMC7683601; doi:10.1038/s41598-020-77366-7)

**Liquid biopsy based on small Extracellular Vesicles predicts chemotherapy response of canine multicentric lymphomas**

Taismara K. Garnica^1^, Jéssika C.C. Lesbon^1^, Ana C.F.CM. Ávila^2^, Arina L. Rochetti^1^, Oscar R.S. Matiz^3^, Roana C.S. Ribeiro^3^, Aline Zoppa⁴, Adriana T. Nishiya⁴, Mirela T. Costa^3^, Andrigo B. de Nardi^3^, David J. Argyle⁵, Ricardo F. Strefezzi^1^, Juliano C. Silveira^2^, Heidge Fukumasu^1^.

¹ Laboratory of Comparative and Translational Oncology (LOCT), Department of Veterinary Medicine, Faculty of Animal Science and Food Engineering, University of Sao Paulo, Pirassununga, Brazil, ZIP

² Laboratory of Molecular Morphophysiology and Development (LMMD), Department of Veterinary Medicine, Faculty of Animal Science and Food Engineering, University of Sao Paulo, Pirassununga, Brazil.

³ Clinical Veterinary Department, College of Agricultural and Veterinary Sciences, São Paulo State University 'Júlio de Mesquita Filho' (UNESP), Jaboticabal, São Paulo, Brazil.

⁴ Veterinary Hospital Anhembi Morumbi, Anhembi Morumbi University, São Paulo, SP, Brazil.

⁵ The Roslin Institute and Royal (Dick) School of Veterinary Studies, The University of Edinburgh, Edinburgh, UK.

Corresponding author: Heidge Fukumasu

E-mail address: [fukumasu@usp.br](mailto:fukumasu@usp.br)

Supplementary table 1. Primers for Oncomir detection by real time PCR.

| Target | Sequence (5'-3') |
| --- | --- |
| bfa-miR-122 | TGGAGTGTGACAATGGTGTTTG |
| bta-let-7a-5p | TGAGGTAGTAGGTTGTATAGTT |
| bta-let-7b | TGAGGTAGTAGGTTGTGTGGTT |
| bta-let-7c | TGAGGTAGTAGGTTGTATGGTT |
| bta-let-7d | AGAGGTAGTAGGTTGCATAGTT |
| bta-let-7e | TGAGGTAGGAGGTTGTATAGT |
| bta-let-7f | TGAGGTAGTAGATTGTATAGTT |
| bta-let-7g | TGAGGTAGTAGTTTGTACAGTT |
| bta-let-7i | TGAGGTAGTAGTTTGTGCTGTT |
| bta-miR-103 | AGCAGCATTGTACAGGGCTATGA |
| bta-miR-106b | TAAAGTGCTGACAGTGCAGAT |
| bta-miR-125b | TCCCTGAGACCCTAACTTGTGA |
| bta-miR-126-5p | CATTATTACTTTTGGTACGCG |
| bta-miR-128 | TCACAGTGAACCGGTCTCTTT |
| bta-miR-132 | TAACAGTCTACAGCCATGGTCG |
| bta-miR-134 | TGTGACTGGTTGACCAGAGTGG |
| bta-miR-135b | TATGGCTTTTCATTCCTATGTGA |
| bta-miR-136 | ACTCCATTTGTTTTGATGATGGA |
| bta-miR-145 | GTCCAGTTTTCCCAGGAATCCCT |
| bta-miR-149-5p | TCTGGCTCCGTGTCTTCACTCCC |
| bta-miR-151-5p | TCGAGGAGCTCACAGTCTAGT |
| bta-miR-155 | TTAATGCTAATCGTGATAGGGGT |
| bta-miR-15a | TAGCAGCACATAATGGTTTGT |
| bta-miR-16b | TAGCAGCACGTAAATATTGGC |
| bta-miR-181d | AACATTCATTGTTGTCGGTGGGT |
| bta-miR-185 | TGGAGAGAAAGGCAGTTCCTGA |
| bta-miR-186 | CAAAGAATTCTCCTTTTGGGCT |
| bta-miR-188 | CATCCCTTGCATGGTGGAGGGT |
| bta-miR-18a | TAAGGTGCATCTAGTGCAGATA |
| bta-miR-190a | TGATATGTTTGATATATTAGGT |
| bta-miR-190b | TGATATGTTTGATATTGGGTT |
| bta-miR-194 | TGTAACAGCAACTCCATGTGGA |
| bta-miR-195 | TAGCAGCACAGAAATATTGGCA |
| bta-miR-196a | TAGGTAGTTTCATGTTGTTGGG |
| bta-miR-197 | TTCACCACCTTCTCCACCCAGC |
| bta-miR-200b | TAATACTGCCTGGTAATGATG |
| bta-miR-200c | TAATACTGCCGGGTAATGATGGA |
| bta-miR-204 | TTCCCTTTGTCATCCTATGCCT |
| bta-miR-205 | TCCTTCATTCCACCGGAGTCTG |
| bta-miR-206 | TGGAATGTAAGGAAGTGTGTGG |
| bta-miR-20a | TAAAGTGCTTATAGTGCAGGTAG |
| bta-miR-210 | ACTGTGCGTGTGACAGCGGCTGA |
| bta-miR-214 | ACAGCAGGCACAGACAGGCAGT |
| bta-miR-22-3p | AAGCTGCCAGTTGAAGAACTG |
| bta-miR-221 | AGCTACATTGTCTGCTGGGTTT |
| bta-miR-222 | AGCTACATCTGGCTACTGGGT |
| bta-miR-24 | GTGCCTACTGAGCTGATATCAGT |
| bta-miR-25 | CATTGCACTTGTCTCGGTCTGA |
| bta-miR-26a | TTCAAGTAATCCAGGATAGGCT |
| bta-miR-26b | TTCAAGTAATTCAGGATAGGTT |
| bta-miR-27a-3p | TTCACAGTGGCTAAGTTCCG |
| bta-miR-27b | TTCACAGTGGCTAAGTTCTGC |
| bta-miR-29b | TAGCACCATTTGAAATCAGTGTT |
| bta-miR-29c | TAGCACCATTTGAAATCGGTTA |
| bta-miR-30b-5p | TGTAAACATCCTACACTCAGCT |
| bta-miR-30c | TGTAAACATCCTACACTCTCAGC |
| bta-miR-423-5p | TGAGGGGCAGAGAGCGAGACTTT |
| bta-miR-488 | TTGAAAGGCTGTTTCTTGGTC |
| bta-miR-92a | TATTGCACTTGTCCCGGCCTGT |
| bta-miR-92b | TATTGCACTCGTCCCGGCCTCC |
| bta-miR-93 | CAAAGTGCTGTTCGTGCAGGTA |
| cfa-miR-1-1 | TGGAATGTAAAGAAGTATGTA |
| cfa-miR-101 | TACAGTACTGTGATAACTGA |
| cfa-miR-106a | AAAGTGCTTACAGTGCAGGTAG |
| cfa-miR-107 | AGCAGCATTGTACAGGGCTAT |
| cfa-miR-10b | CCCTGTAGAACCGAATTTGTGT |
| cfa-miR-125a | TCCCTGAGACCCTTTAACCTGT |
| cfa-miR-133a | TTGGTCCCCTTCAACCAGCTGT |
| cfa-miR-137 | TTATTGCTTAAGAATACGCGT |
| cfa-miR-140 | ACCACAGGGTAGAACCACGGA |
| cfa-miR-141 | AACACTGTCTGGTAAAGATGG |
| cfa-miR-142 | CCCATAAAGTAGAAAGCACTA |
| cfa-miR-143 | TGAGATGAAGCACTGTAGCTC |
| cfa-miR-146a | TGAGAACTGAATTCCATGGGTT |
| cfa-miR-150 | TCTCCCAACCCTTGTACCAGTG |
| cfa-miR-153 | TTGCATAGTCACAAAAGTGA |
| cfa-miR-15b | TAGCAGCACATCATGGTTTA |
| cfa-miR-181a | AACATTCAACGCTGTCGGTGAG |
| cfa-miR-181b | AACATTCATTGCTGTCGGTG |
| cfa-miR-181c | AACATTCAACCTGTCGGTGAGTT |
| cfa-miR-183 | TATGGCACTGGTAGAATTCACT |
| cfa-miR-191 | CAACGGAATCCCAAAAGCAGCT |
| cfa-miR-192 | CTGACCTATGAATTGACAGCC |
| cfa-mir-199 | ACAGTAGTCTGCACATTGGTT |
| cfa-miR-200a | CATCTTACCGGACAGTGCTGGA |
| cfa-miR-202 | TTCCTATGCATATACTTCTTTG |
| cfa-miR-21 | TAGCTTATCAGACTGATGTTGA |
| cfa-miR-215 | ATGACCTACGAATTGATAGACA |
| cfa-miR-218 | TTGTGCTTGATCTAACCATGT |
| cfa-miR-223 | TGTCAGTTTGTCAAATACCCC |
| cfa-miR-224 | CAAGTCACTAGTGGTTCCGTTT |
| cfa-miR-23a | ATCACATTGCCAGGGATTT |
| cfa-miR-29a | TAGCACCATCTGAAATCGGTTA |
| cfa-miR-30a | TGTAAACATCCTCGACTGGAAGC |
| cfa-miR-9 | TCTTTGGTTATCTAGCTGTATGA |
| bta-miR-99b (Housekeeping_1) | CACCCGTAGAACCGACCTTGCG |
| Hm/Ms/Rt T1 snRNA (Housekeeping_2) | CGACTGCATAATTTGTGGTAGTGG |
| RNT43 snoRNA (Housekeeping_3) | CTTATTGACGGGCGGACAGAAAC |

Supplementary table 2. Data on patients of lymphoma and control groups.

|  | Lymphoma group (n = 19) | Control Group (n = 30) | P value |  |
| --- | --- | --- | --- | --- |
| Sex | Male n = 09  Female n = 10 | Male n = 14  Female n = 16 | 0.9617 |  |
| Age | Male  4 – 11 years (mean 7.9)  Female  3 – 14 years (mean 7.7) | Male  1 – 15 years (mean 4.1)  Female  0.5 – 13 years (mean 2.7) | <0.0001 |  |
| Breed | Mixed Breed n = 7  Lhasa Apso n = 2  Labrador Retriever n = 2  Golden Retriever n = 1  Boxer n = 1  West High England terrier n = 1  Pit Bull n = 1  Doberman Pinscher n = 1  Poodle n = 1  American Bully n = 1  White Swiss Shepperd Dog n = 1 | Mixed Breed n = 13  Border Collie n = 3  Shi Tzu n = 2  Labrador Retriever n = 2  Golden Retriever n = 2  Australian Cattle dog n = 1  Lhasa Apso n = 1  Maltese n = 1  Yorkshire n = 1  Pit Bull n = 1  Doberman Pinscher n = 1  White Swiss Shepperd Dog n = 1  German Shepperd dog n = 1 | 0.6524 |  |

Supplementary table 3. Clinical and diagnostic data of lymphoma patients engaged in the study.

|  | *Sample* | *Hospital* | *Sex* | *Age (years)* | *Breed* | *Weight (kg)* | *Cytology* | *IMH* | *PARR* | *Stage* | *Substage* | *CHOP (weeks)* | *Outcome* | *Relapse* | *OS* | *DFI* |
| --- | --- | --- | --- | --- | --- | --- | --- | --- | --- | --- | --- | --- | --- | --- | --- | --- |
| *PD* | *NR_1* | *FCAV* | *F* | *8,0* | *Mixed Breed* | *32,5* | *Large-cell lymphoma* |  |  | *5* | *B* | *4* | *Dead* |  | *24,00* |  |
|  | *NR_10* | *UAM* | *F* | *10,0* | *Mixed Breed* | *33,35* | *Large-cell lymphoma* |  |  | *4* | *B* | *3* | *Dead* |  | *116,00* |  |
|  | *NR_11* | *UAM* | *F* | *11,0* | *West High England Terrier* | *9* | *Large-cell lymphoma* |  |  | *5* | *A* | *4* | *Dead* | *yes* | *171,00* |  |
|  | *NR_13* | *UAM* | *M* | *5,0* | *Lhasa Apso* | *5,7* | *Large-cell lymphoma* |  |  | *5* | *B* | *4* | *Dead* |  | *101,00* |  |
|  | *NR_2* | *FCAV* | *M* | *9,0* | *Mixed Breed* | *12,5* | *Large-cell lymphoma* |  |  | *4* | *B* | *4* | *Dead* | *yes* | *51,00* |  |
|  | *NR_3* | *FCAV* | *M* | *9,0* | *Boxer* | *20* | *Large-cell lymphoma* |  |  | *5* | *B* | *1* | *Dead* |  | *12,00* |  |
|  | *NR_4* | *FCAV* | *M* | *11,0* | *Mixed Breed* | *9,3* | *Large-cell lymphoma* |  |  | *4* | *B* | *4* | *Dead* |  | *78,00* |  |
|  | *NR_5* | *FCAV* | *M* | *9,0* | *White Swiss Shepperd Dog* | *47* | *Large-cell lymphoma* |  |  | *5* | *B* | *8* | *Dead* | *yes* | *115,00* |  |
|  | *NR_6* | *FCAV* | *F* | *7,0* | *Pitbull* | *21,6* | *Large-cell lymphoma* |  |  | *4* | *B* | *7* | *Dead* | *yes* | *228,00* |  |
|  | *NR_7* | *UAM* | *M* | *11,0* | *Pinscher* | *3,15* | *Large-cell lymphoma* |  |  | *4* | *B* | *19* | *Dead* | *yes* | *223,00* |  |
|  | *NR_8* | *UAM* | *F* | *14,0* | *Poodle* | *8,1* | *Large-cell lymphoma* | *DLBCL* |  | *4* | *B* | *12* | *Dead* |  | *248,00* |  |
| *CR* | *R_1* | *FCAV* | *M* | *4,0* | *Labrador Retriever* | *38,3* | *Large-cell lymphoma* |  | *B-cell* | *5* | *B* | *19* | *Alive* |  | *744,00* | *555,00* |
|  | *R_2* | *FCAV* | *F* | *5,0* | *Mixed Breed* | *8,9* | *Large-cell lymphoma* | *DLBCL* |  | *3* | *A* | *19* | *Dead* |  | *440,00* | *286,00* |
|  | *R_3* | *FCAV* | *F* | *6,0* | *Golden Retriever* | *45,5* | *Large-cell lymphoma* | *DLBCL* |  | *3* | *A* | *19* | *Alive* | *yes* | *614,00* | *217,00* |
|  | *R_4* | *FCAV* | *F* | *3,0* | *American Bully* | *26,5* | *Large-cell lymphoma* |  | *B-cell* | *4* | *B* | *19* | *Dead* | *yes* | *328,00* | *90,00* |
|  | *R_5* | *FCAV* | *F* | *6,0* | *Mixed Breed* | *18,5* | *Large-cell lymphoma* | *DLBCL* |  | *3* | *A* | *19* | *Alive* |  | *501,00* | *347,00* |
|  | *R_6* | *FCAV* | *M* | *6,0* | *Lhasa Apso* | *6,5* | *Large-cell lymphoma* | *DLBCL* |  | *4* | *A* | *19* | *Dead* | *yes* | *461,00* | *314,00* |
|  | *R_7* | *FCAV* | *F* | *7,0* | *Mixed Breed* | *20,9* | *Large-cell lymphoma* |  |  | *3* | *A* | *19* | *Alive* |  | *837,00* | *669,00* |
|  | *R_8* | *UAM* | *M* | *7,0* | *Labrador Retriever* | *45,5* | *Large-cell lymphoma* |  |  | *4* | *B* | *19* | *Alive* |  | *660,00* | *533,00* |

*Legend: Staging according to WHO (Owen 1980), Cytology according to Ponce et al 2010; Immunohistochemistry according to Langner et al 2014; PARR according to Valli et al 2011.*

Supplementary figure 1. Western blot membrane used for anti-CD9 (upper part of the membrane) and anti-CYT C (lower part of the membrane).


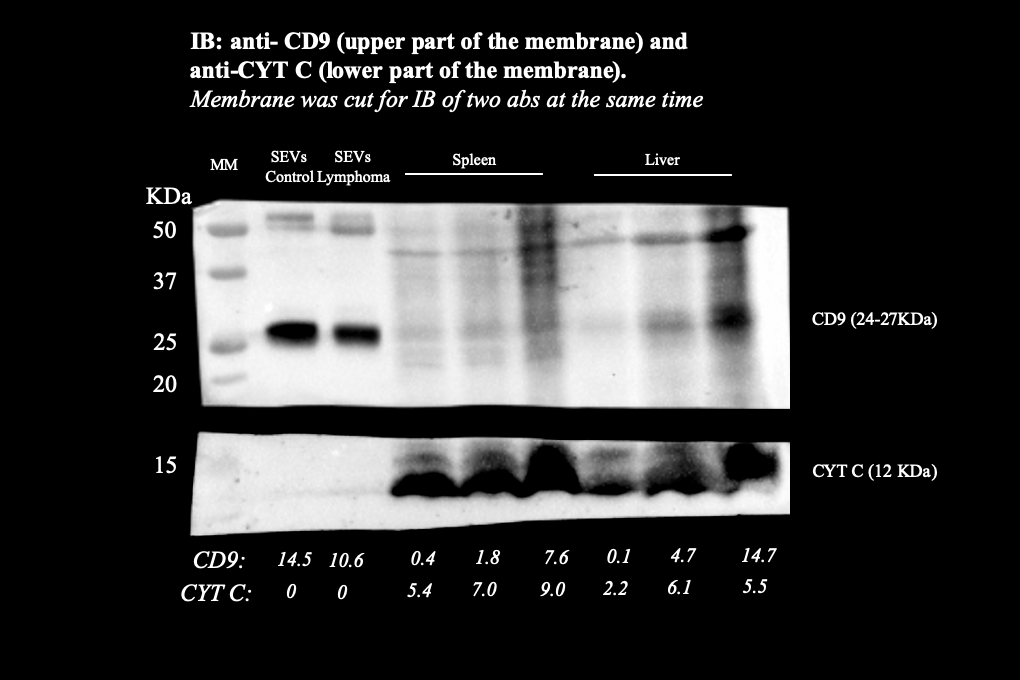

Supplement: Supplementary file 1 — Supplementary Tables. [file 41598_2020_77366_MOESM1_ESM.docx]
